# Clamping force prediction based on deep spatio-temporal network for machining process of deformable parts

**DOI:** 10.1038/s41598-023-33666-2

**Published:** 2023-04-28

**Authors:** Enming Li, Jingtao Zhou, Changsen Yang, Mingwei Wang, Shusheng Zhang

**Affiliations:** 1 School of Mechanical Engineering, Northwestern Polytechnical University, Xi’an, 710072 China; 2 Engineering Research Center of Advanced Manufacturing Technology for Aero Engine (Northwestern Polytechnical University), Ministry of Education, Xi’an, 710072 China

**Keywords:** Engineering, Mechanical engineering

## Abstract

As an important component of the machining system, the influence of fixtures on the machining deformation of the workpiece cannot be ignored. By controlling the clamping force during the machining process is an effective means to suppress or improve the machining deformation. However, due to the dynamic coupling of part geometry, clamping method, manufacturing process and time-varying cutting forces, it is difficult to obtain accurate clamping forces, which hinders the realization of fixture-based deformation control. In this paper, the variation of clamping force is considered as the response of the joint action of cutting force and other working conditions in spatial and temporal terms, and a clamping force prediction method based on deep spatio-temporal network is proposed. The part geometry model is first parameterized based on voxels, after which the cutting forces are dynamically correlated with the clamping forces in spatial and temporal terms. Then, a convolutional network was designed to capture the spatial correlation between the working conditions such as cutting force and clamping force, and a gated recurrent cell network to capture the temporal correlation to predict the clamping force during machining. Finally, an experiment of milling a cylindrical thin-walled part illustrates the effectiveness of the proposed method.

## Introduction

Machining deformation is a common issue in the machining of thin-walled parts, such as magazines, blades and other parts in the aerospace industry, which not only seriously affects the machining accuracy but even leads to the scrapping of the parts^1,2^. The machining deformation of the part is generated mainly for two reasons, one is due to material removal, the deformation caused by the redistribution of residual stress inside the part, and the second is the deformation caused by external loads (mainly clamping and cutting forces) during the machining process^3^. For the second point above, the key means to avoid machining deformation caused by external loads is through dynamic adjustment of the clamping force or optimization of the fixture system layout.

As an important part of the overall machining system, the fixture is a sophisticated subsystem that provides accurate positioning of the part in the working space in addition to rigidly fixing and supporting the part^4^. The setting of the clamping force is one of the main factors affecting the deformation during machining^5^, especially when machining thin-walled parts, a larger clamping force can lead to excessive elastic deformation of the part and cause large dimensional errors, while a smaller clamping force cannot adequately restrain the part. Therefore, the accurate acquisition of clamping force during machining is the key to study the overall force on the part, and to further suppress machining deformation by adjusting the fixture.

Accurate prediction of clamping force, for one thing, offers input to some part processing deformation prediction and control methods, such as part deformation control methods based on pre-deformation^6^, or part deformation prediction methods based on the finite element method^7^. In the actual machining process, for some machining scenarios with adjustable fixtures, the clamping force can be predicted to adjust the fixture contact point or clamping force in advance to balance the overall force on the part, or even release the machining deformation, and then eliminate the part deformation through subsequent machining, so as to improve the machining efficiency while ensuring the part machining quality. However, conventional clamping force settings are often based on manual experience with large uncertainties, and little consideration is given to dynamic adjustment of the clamping force according to the machining process. Further, the change of clamping force is affected by a combination of cutting parameters, cutting force, clamping position and other factors in the machining process, and these factors show a complex spatio-temporal coupling relationship with the change of working conditions. For example, the cutting force at the current cutting contact point will cause changes in the clamping force of the part. Similarly, the fixture will also respond to the cutting force, and the two show a correlation in the geometric space of the part. Further, as machining proceeds, the cutting force action point changes with the tool trajectory, which in turn also causes the clamping force to change, and the two show a temporal correlation, leading to a difficult prediction of the clamping force.

Traditional methods for predicting forces on machining processes. There are two main types of traditional force prediction for machining processes, which are based on cutting mechanism, and finite element simulation.The mechanical analysis modeling mainly refers to the theoretical analysis of the forces and related parameters in the processing process, to establish the corresponding theoretical numerical model^8,9^. And the finite element simulation method refers to offline machining simulation prediction using commercial finite element simulation software^10–12^.

Zhang et al. developed a kinematic model of the part-fixture system using the principle of minimum modulus^13^, and calculated the contact forces between the fixture components and the parts. Wei et al.^14^ proposed a milling force prediction model for five-axis flat end milling considering arbitrary feed direction and tool-part contact. Wang et al.^15^ calculated the undeformed chip thickness based on the instantaneous feed rate of the cutting edge of the part, followed by the discretization of the solid to determine the tool engagement, and finally the milling force was obtained by calibrating the specific force coefficient. Liu et al.^16^ used a finite element based method for milling error prediction, and a mathematical model of the actual cutting thickness was developed to obtain more accurate cutting force profiles and lower milling deformation errors, taking into account the deflection feedback of the tool part system. Teramoto et al.^17^ evaluated the deformation of parts under different clamping sequences, using locally measured strains and clamping simulations, and used the response surface method to estimate clamping forces and part deformation. Other researchers have viewed force identification or load distribution identification as an inverse problem, such as Liaghat et al.^18^ modeled the inverse problem of load distribution identification as an optimization problem, proposed an efficient iterative inverse process for identifying the load distribution leading to a specific crack expansion path in fractured members. Xu et al.^19^ proposed a novel inverse method based on two different objective functions for identifying the unknown elastic constants and boundary conditions of three-dimensional hyperelastic materials. Nevertheless, the mechanistic analysis clearly indicates which factors affect the complex physical process. For example, the clamping force is considered as a contact force between the part and the clamping element, which from the point of view of mechanical analysis is the combined force of the stresses applied to the contact surface, and Li et al.^20^ point out that the contact stresses are influenced by the axial load in their analysis of the sealing effect. Similar studies give theoretical support for analyzing which factors have a quantitative relationship with the clamping force.

The above-mentioned traditional methods for predicting forces in machining processes rely on input parameters that are difficult to obtain or estimate, such as tool-workpiece contact and workpiece-fixture contact, which are very complicated to model or simulate. In addition, theoretical mechanical analysis and finite element simulations perform some simplifications that differ from the actual situation in order to be able to solve and converge, and these may lead to less adaptability of the prediction model to different machining processes or scenarios.

Data-driven force prediction method. Today, the development of sensors and online monitoring technologies has made it possible to monitor the actual state of the manufacturing system in real time^21^. Displacement sensors or strain gauges, for example, have been used in fixtures and clamping elements to monitor clamping and reaction forces, and this has led to the design of dynamically adjustable fixtures^3,22^ and floating clamping methods based on real-time displacement monitoring data^13^. Compared to sensors and online monitoring, the rapid development in the field of computer science, such as machine learning and deep learning, provides new data-driven ideas for discovering patterns in machining processes from these monitoring data^23^, especially for complex, dynamic, and even chaotic manufacturing processes embodying great advantages. Applications such as those of the authors' research group in tool condition monitoring^24,25^, and other researchers in machining accuracy prediction^26^ or machining deformation prediction problems^27^.

Xu et al.^28^ proposed a cutting force prediction model, ForceNet, which combines basic physical prior knowledge with a structured neural network to represent complex tool-part meshing variations with grayscale images to achieve prediction of offline cutting forces. Wang et al.^29^ established a transfer network for the cutting force prediction task based on the data obtained from simulations, with the simulated data representing the source domain and the experimental data representing the target domain. The neural network was trained to achieve the prediction of cutting force. In addition to the methods mentioned above, researchers have attempted to use some mechanistic and data-driven fusion of force analysis method. For example, Liu et al.^30^ constructed a clamping force optimization method considering friction effects, combining finite elements with genetic algorithms, which can achieve fixture layout and clamping force optimization with a small number of finite element calculations. Wang et al.^31^ proposed a digital dual-drive clamping force control method, which can dynamically adjust the clamping force according to the clamping deformation during the clamping force control process, by combining finite element simulation and deep learning network to establish a virtual space model. However, existing research has been more focused on optimizing the clamping force as a fixed load, or on controlling the clamping force for a specific part and a specific clamping method. Clamping forces tend to fluctuate and vary during actual machining, and the lack of real-time, accurate clamping force information hinders better decision making during the machining of thin-walled parts.

The clamping force, as an important external load during the machining of thin-walled parts, is critical in terms of its spatial distribution and temporal sequence from the perspective of the load identification problem. Inspired by this, aiming at the problem of clamping force prediction in thin-walled parts machining process, this paper proposed a clamping force prediction method based on deep learning from the perspective of the spatio-temporal correlation between each working condition and clamping force in machining process. In summary, a deep spatio-temporal network-based clamping force prediction method is proposed in this paper, the core of which is to model and solve the spatio-temporal correlation between cutting force and clamping force in the part geometry space.

This paper is structured as follows. The framework of the clamping force prediction method is given in Sect. 2. Section 3 describes the part geometry model parameterization and the process of dynamic correlation of cutting and clamping forces in the spatial and temporal context. Section 4 focuses on the construction process of a deep spatio-temporal network for clamping force prediction. Experimental validation and discussion are carried out in Sect. 5. Finally, a summary of this study is presented in Sect. 6.

## Overview of the proposed approach

In this paper, a new method for predicting clamping force based on deep spatio-temporal networks is proposed. The complex relationship between cutting force and clamping force is regarded as the process of spatio-temporal correlation between the two on the part geometry. The part geometry model is used as the spatial medium for the interaction of cutting and clamping forces, and it is parameterized as the spatial properties of cutting and clamping forces. Then, the cutting force with spatial trajectory information, and cutting parameters are used as input, and the clamping force with spatial coordinate information is used as output , and the clamping force prediction model based on spatio-temporal network is established. The prediction of the clamping force during machining can provide the basis for subsequent deformation control by changing the clamping state or optimizing the fixture layout.

**Figure 1:**
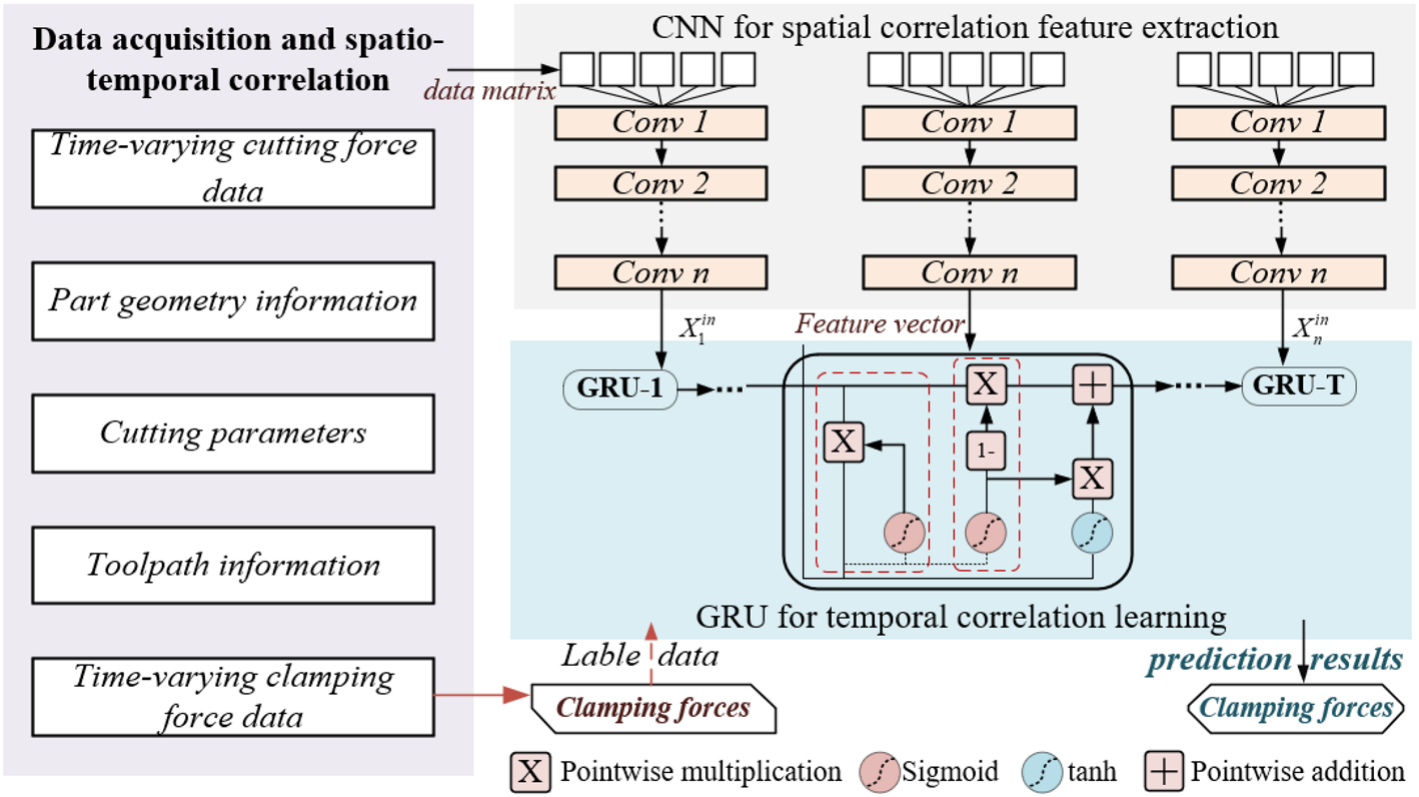
The framework of the clamping force prediction based on deep spatio-temporal network.

The basic procedure of the proposed method is illustrated in Fig. 1. It consists of three main parts: data acquisition and spatio-temporal correlation, the spatial correlation modeling, and the temporal correlation modeling to achieve the final clamping force prediction. A brief description of each part is given in this section.

The working conditions affecting the clamping force variation during the machining process show diverse, time-varying and interrelated characteristics. The simple fusion of the relevant conditions will lose the key interaction information between the conditions and affect the effectiveness of the subsequent prediction model. Considering that the key problem of load identification lies in its spatial distribution and time sequence characteristics, the temporal and spatial correlation of the conditions affecting the change of clamping force is firstly carried out.

1. Data acquisition and spatio-temporal correlation

In this paper, the geometry of the part is both the spatial carrier and the medium of action for the interaction of the cutting and clamping forces. This means that information on the spatial location of the cutting and clamping forces on the part (e.g. cutting trajectory and clamping point location) is required. Therefore, the part geometry model is used as the basis for the spatio-temporal correlation of factors such as clamping force and cutting force. Firstly, the geometric model is parameterized, and the parameterized geometric information is used as the spatial attributes of cutting force and clamping force; then, the cutting force and clamping force are correlated under a uniform time interval using the tool trajectory.

(2) Spatial correlation feature extraction

Furthermore, since cutting forces and clamping forces are spatially correlated, meaning that they interact with each other in the part geometry space, this situation can be effectively handled by a convolutional neural network (CNN), which has a natural ability to capture spatial dependence in its structure^32^. In view of this, this paper implements spatial correlation learning between cutting force and clamping force by constructing a multilayer CNN.

(3) Temporal correlation learning

Then, the spatial relationship between cutting force and clamping force over time is considered, mainly due to the fact that cutting force acts on different cutting points with the tool trajectory, and different cutting points have different locations from the clamping point, which have different magnitudes of influence on the clamping force. Therefore, the position information is taken as a component of the two types of forces, and then the cutting force and clamping force with position information at different moments are considered as a time slice, and the temporal correlation between the two is established through a gated recurrent unit (GRU) network that can capture the temporal relationship.

As shown in Fig. 1, the whole deep spatio-temporal network is trained in a joint manner. The actual data of clamping force is used as labels to input into the GRU network, and the GRU network is trained together with the above input spatial features to achieve the prediction model of clamping force. In fact, multiple forces at different clamping locations can be collected, and one part can be used as input to the CNN network for spatial correlation relationship learning, and the other part can be input to the GRU network as label values for prediction model training, and in turn both can be verified with each other.

## The spatial and temporal correlation process between cutting force and clamping force

### Diagram of spatio-temporal correlation

**Figure 2:**
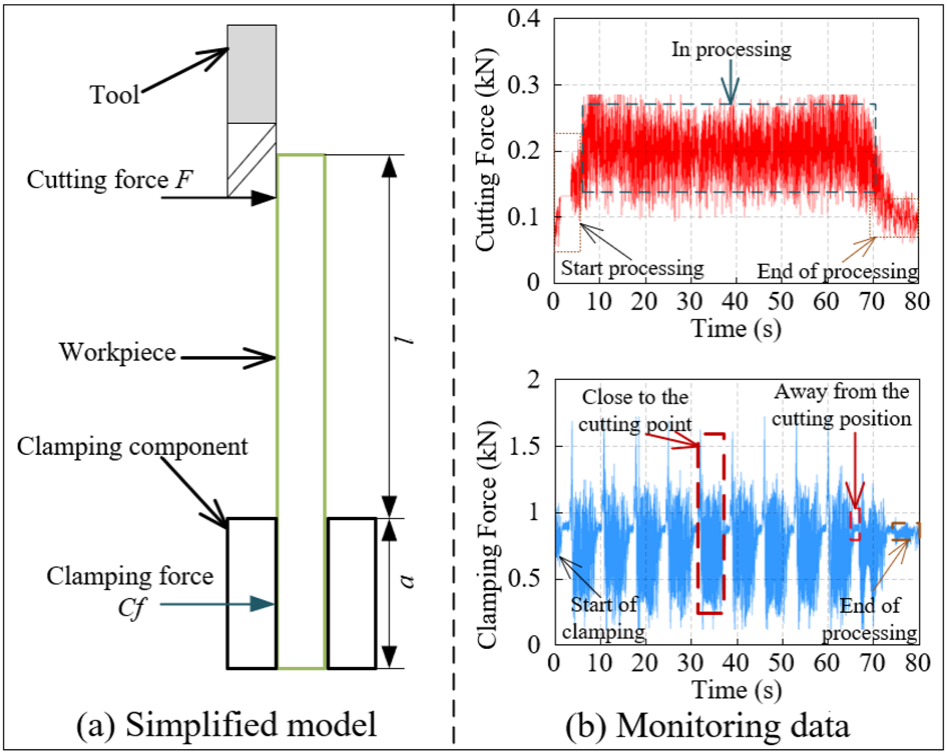
Explanation of spatio-temporal correlations.

The clamping force is often estimated based on the known and simulated cutting forces in order to ensure that the part does not loosen during machining. The commonly used method is based on the moment balance between the cutting force and the clamping force. For example, the workpiece-fixture system is simplified to a cantilever, as shown in Fig. 2a below, where the cutting force acts on the free end and has a rotational effect on the cantilever. At the same time, the clamping force generates a clamping effect to oppose this rotation.

According to the torque balance, we have *Cf*·*a*-*F*·*l* = 0. This means that when the tool applies a force to the workpiece, the application point will respond to the workpiece. At the same time, this response will be transferred to the fixture through the workpiece. This suggests that cutting and clamping forces are correlated through the part geometry, which provides a potential or novel idea for monitoring or predicting clamping forces during cutting. As shown in Fig. 2b, the spatial relationship between the cutting force and the clamping force means that the cutting force at different cutting positions acts on the clamping force in different magnitudes. The temporal correlation between them means that the change of cutting force causes the change of clamping force as the machining progresses, and the spatial correlation between cutting force and clamping force at different cutting positions is then considered as a time slice.

**Figure 3:**
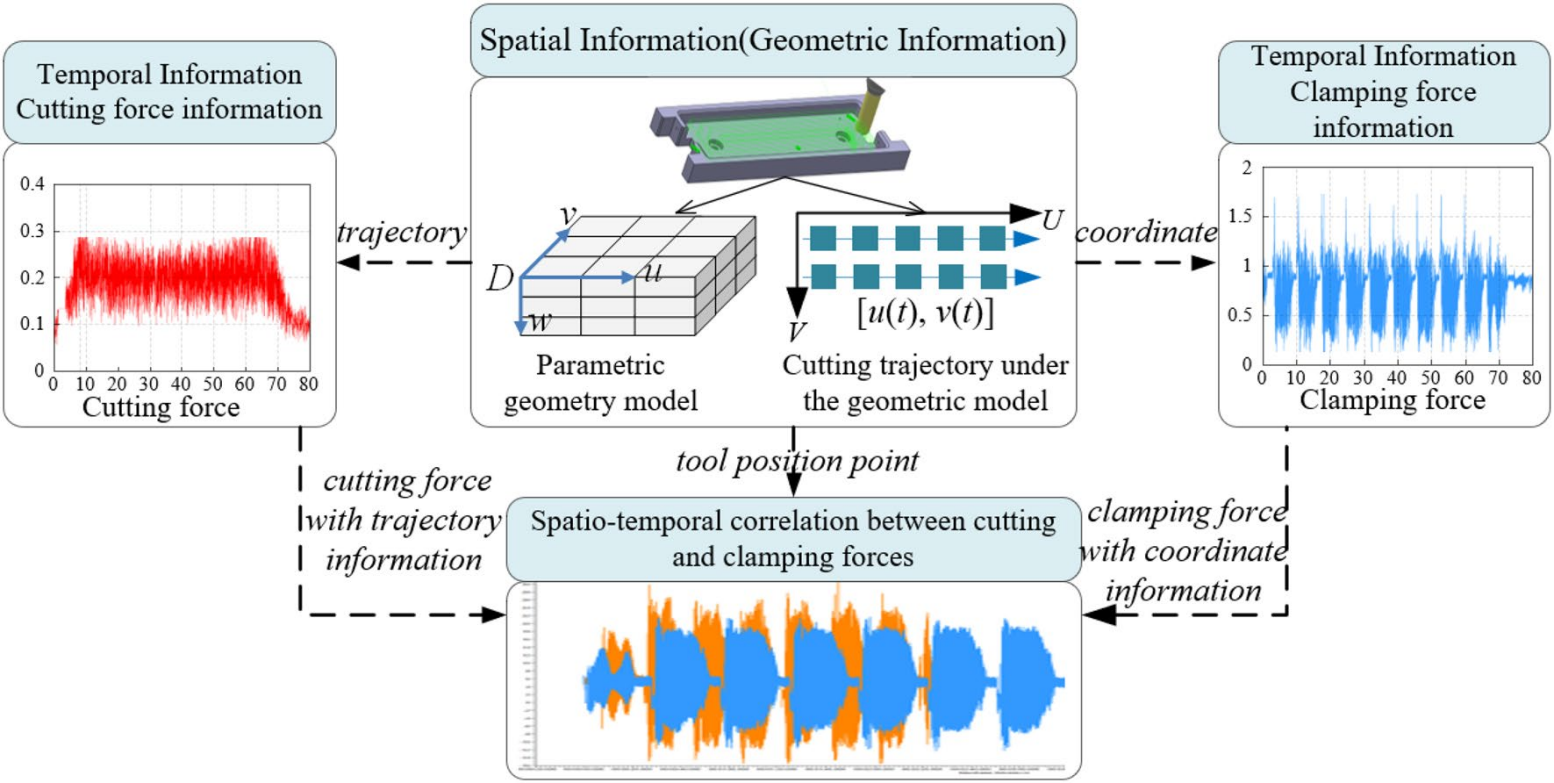
Schematic diagram of spatio-temporal correlation between cutting and clamping forces.

Considering the complexity of the workpiece geometry and the uncertainty of the clamping position, we extend the dynamic influence relationship between the cutting force and clamping force in spatial terms to the whole workpiece geometry space during machining. The main point of this section is to correlate two different types of forces (mainly different positions of application and patterns of change of the forces) in a synchronized manner. The geometric model of the part is first parameterized as the spatial basis for the spatio-temporal correlation of the cutting force and the clamping force; then, the cutting force and the clamping force are correlated spatio-temporally using the tool trajectory of the cutting process. As shown in Fig. 3 below.

As can be seen from the above figure, firstly, the geometric model of the part is parameterized, and the parameter information of its position on the plane after parameterization is used as the spatial basis for the association of cutting force and clamping force. Secondly, the corresponding tool trajectory information is discrete into tool position points, and the cutting force is associated with the tool trajectory according to the tool position point information, and the clamping force is associated with the fixed coordinate information on the two-dimensional plane. Finally, the information of two types of unsynchronized forces is associated through the time interval of adjacent tool position points , so as to realize the association of the two in spatial and temporal terms.

### Voxel-based geometry model parameterization

In the machining process, cutting parameters, cutting forces, etc. can be seen as the input or excitation of the whole machining system, and the clamping force and geometric state of the part can be seen as the output or response of the system^33^. Moreover, the geometric model parameterization is the spatial basis for the spatio-temporal interaction of cutting forces and clamping forces, therefore, the geometric model of the part needs to be parameterized first. Inspired by the deformation mapping method in constructing the process model, the 3D solid model of the part is represented in the parameter space as a variable depth voxel model. In order to obtain the location parameters of the depth-varying voxel model on the part surface, the 3D solid is first mapped from the physical domain to the parametric domain using the mesh generation method, and the 3D model is parameterized in the parametric domain.

The 3D geometric model is represented by the spatial discretization method of 3D deformed surface entities, including the variable-depth voxels in UVW parameter space, the surface entities in XYZ physical space, and the spatial mapping relationship between them, which can be expressed as:[*x*, *y*, *z*] = [*x*(*u*,*v*,*w*), *y*(*u*,*v*,*w*), *z*(*u*,*v*,*w*)]. Then the part machining process is represented by the depth-varying voxel removal sequence.

**Figure 4:**
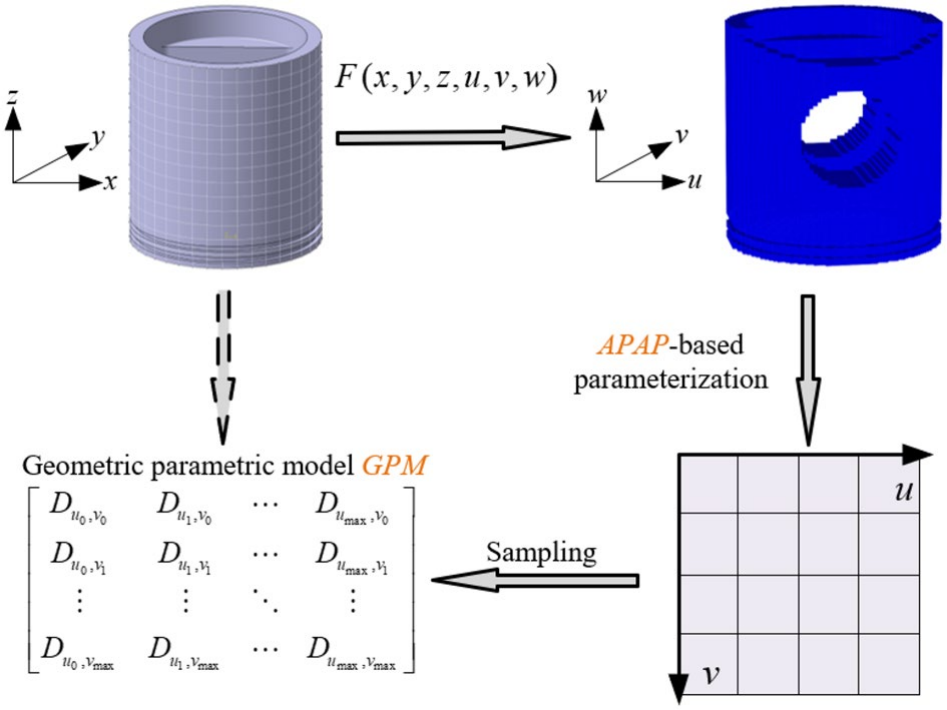
Geometry model parameterization.

To facilitate obtaining information on the relative position of the cutting and clamping forces on the part geometry, the part is angularly mapped from the X–Y plane to the bottom plane. Figure 4 below shows an example of the parameterization process of a geometric model of a cylindrical thin-walled part.

The general method of parameterizing the 3D model requires mesh production techniques to achieve it. Hyperfinite interpolation is a widely adopted algebraic mesh generation method whose mesh checker can be directly controlled and computationally efficient^34^. To illustrate the proposed method, first define the mapping relationship between the Cartesian coordinates (*x*,*y*,*z*) of the physical domain entity model and the corresponding parameter coordinates (*u*,*v*,*w*) of the parameter domain as :

1 $$ F(x,y,z,u,v,w) = \left[ {\begin{array}{*{20}c} {F_{1} (x,y,z,u,v,w)} \\ {F_{2} (x,y,z,u,v,w)} \\ {F_{3} (x,y,z,u,v,w)} \\ \end{array} } \right]{\kern 1pt} {\kern 1pt} {\kern 1pt} {\kern 1pt} {\kern 1pt} {\kern 1pt} {\kern 1pt} {\kern 1pt} {\kern 1pt} {\kern 1pt} {\kern 1pt} {\kern 1pt} (u,v,w) \in [0,1] $$

where *F* is the mapping transformation function.

Here Hermite interpolation is used as a morphing mapping model, and the mapping process in the physical domain in the Z direction is illustrated as an example, with two mapping functions in the Z direction as follows:

2 $$ \begin{gathered} F(x,y,z,u,v,0) \leftrightarrow f_{1} (u,v) \hfill \\ F(x,y,z,u,v,1) \leftrightarrow f_{2} (u,v) \hfill \\ \end{gathered} $$

where the symbol ‘$\leftrightarrow$’ represents a one-to-one mapping relationship, *f*_1_ and *f*_2_ are the initial and finished surface of a machining task, and the values of the *w* parameters for the two corresponding surfaces in the z-direction are *w*_1_ = 0 and *w*_2_ = 1.

Then, the geometric model after hermite hyper-interpolation can be represented in the parameter space as a variable depth voxel model *D*.

3 $$ D = \{ D(u,v,w)\} $$

where [*u*, *v*] is the position parameter on the surface of the part, and *w* is the variable depth parameter in the range of [0,1].

Since the points on the two-dimensional plane may not be uniform, they cannot be represented by a matrix. The two-dimensional array expression of the geometric model can be obtained by uniform sampling in the two-dimensional parameter domain according to the *n* × *n* requirement. Then, for the model *D* in the parametric domain, after parameterization it can be represented in the two-dimensional plane as a geometric parameterized model *G*_*uv*_.

4 $$ G_{uv} = \left[ {\begin{array}{*{20}c} {D_{{u_{0} ,v_{0} }} } & {D_{{u_{1} ,v_{0} }} } & \cdots & {D_{{u_{\max } ,v_{0} }} } \\ {D_{{u_{0} ,v_{1} }} } & {D_{{u_{1} ,v_{1} }} } & \cdots & {D_{{u_{\max } ,v_{1} }} } \\ \vdots & \vdots & \ddots & \vdots \\ {D_{{u_{0} ,v_{\max } }} } & {D_{{u_{1} ,v_{\max } }} } & \cdots & {D_{{u_{\max } ,v_{\max } }} } \\ \end{array} } \right] $$

The part geometry model is divided into voxels according to an equally spaced mesh, where the corresponding index numbers are denoted as (*i*, *j*). Further, the specific values of *i*, *j*, such as 0, 1, … , max refers to the node number of the corresponding plane. Here the coordinates of the points on the 2D plane can be expressed as (*u*_*i*_, *v*_*j*_).

### Spatio-temporal correlation between cutting force and clamping force

From the perspective of geometric simulation of the cutting process, we know that at each moment, the contact points of the cutting trajectory on the tool and the part are on the circular projection of the tool, so that each point of the cutting trajectory mapped on the part can be associated with the tool motion. Therefore, the tool trajectory and part geometry are used here to correlate the cutting and clamping forces in spatial and temporal terms.

Specifically, the cutting force is applied to the part with the tool trajectory, and the magnitude of the cutting force and the location of the action point both change with time. Restricted by the position of the fixture, the clamping force has been constant in its position of action, although the magnitude changes with time. In other words, the cutting force and clamping force have spatial and temporal inconsistency when acting on the part together in the machining process, which is mainly reflected in the inconsistency of the change law of the two over time and the dynamic correlation of the action position in the geometry of the part.

Based on the results of the geometric parameterization model in the previous section, the definition of the cutting force and the clamping force are given first.

5 $$ l\_F(t) = (u(t),v(t),F(t)) $$

where *l*_*F*(*t*) represents the cutting force with coordinate information, the tool cutting sequence *p*_*uv*_(*t*) = (*u*(*t*), *v*(*t*)) are known, and *F*(*t*) is the cutting force that varies with time, i.e., the position and magnitude of the cutting force acting on the part vary with time. Similarly, the clamping force can be expressed as:

6 $$ l\_Cf(t) = (u_{Cf} ,v_{Cf} ,Cf(t)),{\kern 1pt} {\kern 1pt} (u_{Cf} ,v_{Cf} ) \in {\kern 1pt} {\kern 1pt} \{ (u_{i} ,v_{j} )\} $$

where *l*_*Cf*(*t*) is the clamping force with coordinate information, *u*_*Cf*_ and *v*_*Cf*_ are the position coordinates of the clamping points on the two-dimensional plane of the part. *Cf*(*t*) is a time-varying clamping force, i.e. the clamping force acts on the part at a constant position, while the magnitude varies with time. Since the clamping and workpiece contact points are small relative to the part geometry model and can be approximated as point contact, the coordinates of the point on the 2D plane can be used as fixed coordinates of the clamping force application position.

Further, considering that both cutting force and clamping force are changing in real time, it is necessary to unify them into a certain time interval for analysis before establishing the time-series spatial relationship between cutting force and clamping force. Naturally, the time interval of adjacent tool points on the cutting trajectory can be used for analysis, which is due to the relatively fixed time interval of adjacent tool points, and the spacing is short enough not to ignore the cutting force change information, the actual cutting force signal is relatively easy to obtain and the sampling frequency can be pre-set and convenient to calculate. The tool position points are obtained by discrete continuous cutting trajectory, and the distance between two tool position points can be an integer number of tool rotations in one week, which is set here as 1 tool rotation in one week, i.e. unit feed. Here, the tool position point can be expressed as:

7 $$ TP = \{ TP_{1} ,{\kern 1pt} TP_{2} ,{\kern 1pt} ...,{\kern 1pt} TP_{p} \} ,{\kern 1pt} {\kern 1pt} {\kern 1pt} {\kern 1pt} {\kern 1pt} {\kern 1pt} {\kern 1pt} {\kern 1pt} {\kern 1pt} {\kern 1pt} p \in N $$

The subscript *p* in the above equation is the integer number of tool points *TP* obtained after discretizing the tool trajectory (*u*(*t*),*v*(*t*)). Assuming that the distance between adjacent tool points *TP*_*k-1*_ and *TP*_*k*_ is *Δs*_*k*_, the cutting speed of the tool at the tool point *TP*_*k*_ is *v*_*k*_, and the time interval is *interval*_*k*_ = *Δs*_*k*_/*v*_*k*_. 
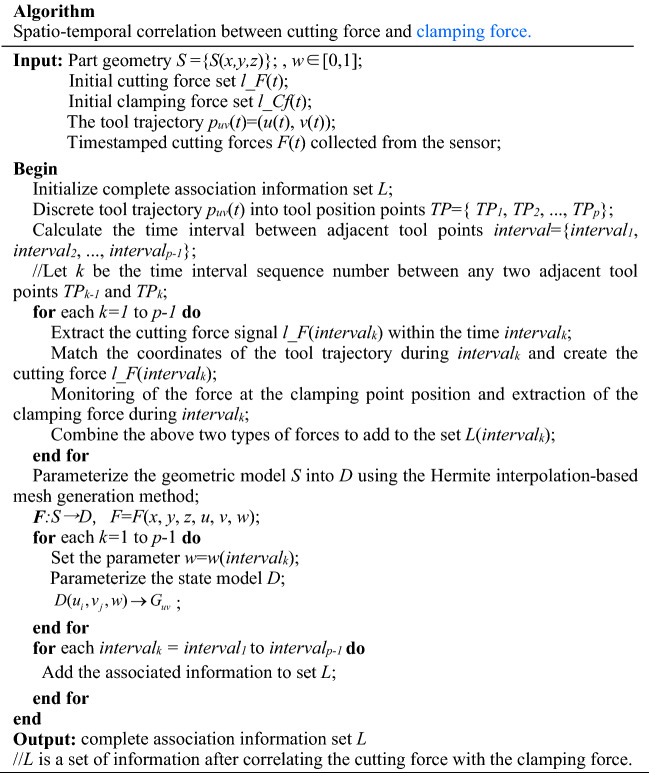


Two types of forces with inconsistent spatial and temporal variations are dynamically correlated through the coordinates of the part in the plane and a uniform time interval, which can better describe the process of spatial and temporal correlation between the actual cutting force and the clamping force on the part.

## Deep spatio-temporal networks for clamping force prediction

### Spatial correlation learning

In this section, CNN networks are mainly used to extract the spatial correlation features between cutting and clamping forces. The main module of the CNN network is a convolutional layer, where a set of weights called a filter bank is used to connect the upper layer to the features and to extract features with highly correlated data. The subsequent pooling process is similar to data compression, and the main purpose is to reduce the dimensionality of the data features and the computational effort of the parameters by performing down-sampling operations on the neurons of the convolutional layer.

**Figure 5:**
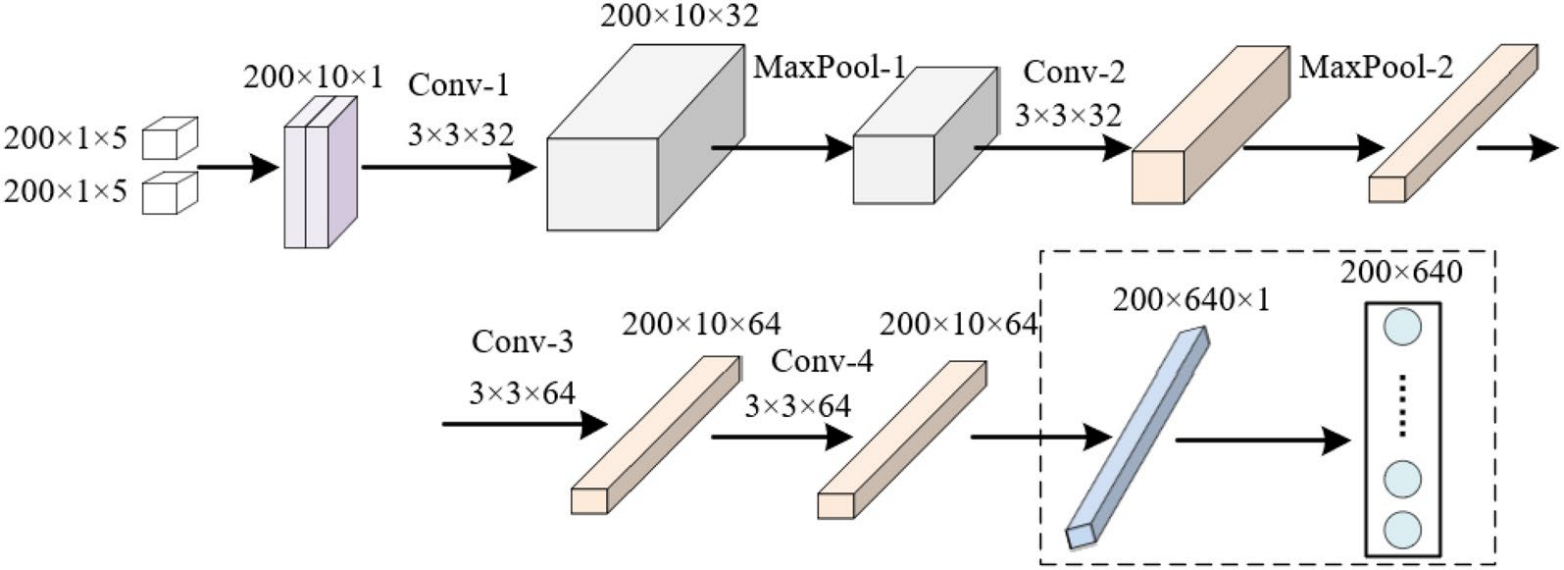
CNN-based spatial correlation features extraction module.

Considering that CNN networks can process small image blocks without the pooling operation^35^, the time-varying cutting force and clamping force data are considered as small pixel images (i.e., small image blocks) and the feature extraction model does not perform the corresponding pooling operation after the convolution operation. The following is an example of the spatial correlation feature extraction process using milling process data, mainly cutting force and clamping force data. The network structure of the spatial correlation learning model is shown in the Fig. 5 below.

The data set* L* of the machining process after the spatio-temporal correlation in the previous section is formed into a 200 × 10 data matrix after the time interval sampling *interval*_*k*_. The cutting force *l*_*F*(*t*) is a 1 × 1 × 5 tensor which is filled by two coordinate information on the plane and three-way force information. Similarly, the clamping force *l*_*Cf*(*t*) can be considered as a 1 × 1 × 5 tensor which is filled by two fixed coordinate information on the plane as well as three-way force information. Then, there are approximately 200 such force information for each time interval (not enough are supplemented with zeros), so that the force information in each time interval can be regarded as a 200 × 1 × 5 tensor. Therefore, the cutting force and the clamping force then have the same matrix size and can be combined into a 200 × 1 × 10 tensor.

Here, a total of 64 feature matrices of size 200 × 10 are obtained after four convolution operations using CNN networks, and then pulled into a 200 × 640 matrix as the final clamping force and cutting force spatial correlation features. First, the original data is preprocessed to obtain the initial data matrix *D*_*T*_. *D*_*T*_ is convolved with 32 convolution kernels of size 3 × 3 × 1 to obtain a feature matrix of size 200 × 10 × 32. Then, the extracted feature matrix is convolved with the same size and number of convolution kernels to obtain a new feature matrix of size 200 × 10 × 32. After that, the previous feature matrix is convolved twice with 64 convolution kernels of size 3 × 3 × 1 to obtain a feature matrix of size 200 × 10 × 64. Finally, it is stretched into a feature matrix of size 200 × 640 as the spatial correlation features of clamping force and cutting force.

Since the final prediction is made for the clamping force, the spatio-temporal network as a whole is trained jointly. The training process of the CNN is given briefly here first. The backward transfer process of the error consists of two stages, firstly, the error is backward transferred in the GRU network, and then it continues to be backward transferred in the CNN network. Considering that only convolutional layers exist in the network structure of the adopted spatial associative learning, the parameter update computation process is given as follows. Forward calculation process (layer *l-*1 to layer *l*):

8 $$ a^{l} = g\left( {z^{l} } \right) = g(a^{l - 1} * w^{l} + b^{l} ) $$

where *a*^*l*^ is the output of the *l*th convolutional network during forward propagation; *g* is the ReLU excitation function; *z*^*l*^ is the output of the *l*th layer after the convolution operation; *a*^*l-*1^ is the output of layer *l*-1 during forward propagation; *w*^*l*^ and *b*^*l*^ are the weights and biases of the *l*th layer, respectively, which are the parameters to be learned.

Reverse propagation process (layer* l* + 1 to layer *l*):

9 $$ \delta^{m,l} = \delta^{m,l + 1} \cdot \frac{{\partial z^{l + 1} }}{{\partial z^{l} }} = \delta^{m,l + 1} *rot180(w^{l + 1} ) \circ g^{^{\prime}} (z^{m,l} ) $$

where *m* is the *m*th sample in the total number of *M* samples; *δ*^*m*,*l*^ is the gradient error of the *m*th sample at the *l*th convolution layer; *δ*^*m*,*l*+1^ is the gradient error of the *m*th sample at the *l* + 1th convolutional layer; *rot180* refers to rotating the matrix by 180°; ○ is the Hadamard product.

Then the weights and biases are updated by the following equation.

10 $$ \begin{gathered} w_{new}^{l} = w^{l} - \eta \sum\limits_{m = 1}^{M} {(a^{m,l - 1} } ) * \delta^{m,l} \hfill \\ b_{new}^{l} = b^{l} - \eta \sum\limits_{m = 1}^{M} {\delta^{m,l} } \hfill \\ \end{gathered} $$

where *w*^*l*^_*new*_ is the updated weight of the *l*th convolutional layer; *η* refers to the learning rate, which is usually taken between (0,1); *b*^*l*^_*new*_ is the updated bias of the *l*th convolutional layer.

Then, the clamping force data *Cf *_*T*+*t*_ at the moment T + t is used as the label, and the parameters in the model are updated and adjusted using the error backward transfer principle, and the final 200 × 640 data matrix is the spatio-temporal correlation feature of the predicted samples at time T.

### Temporal correlation learning

**Figure 6:**
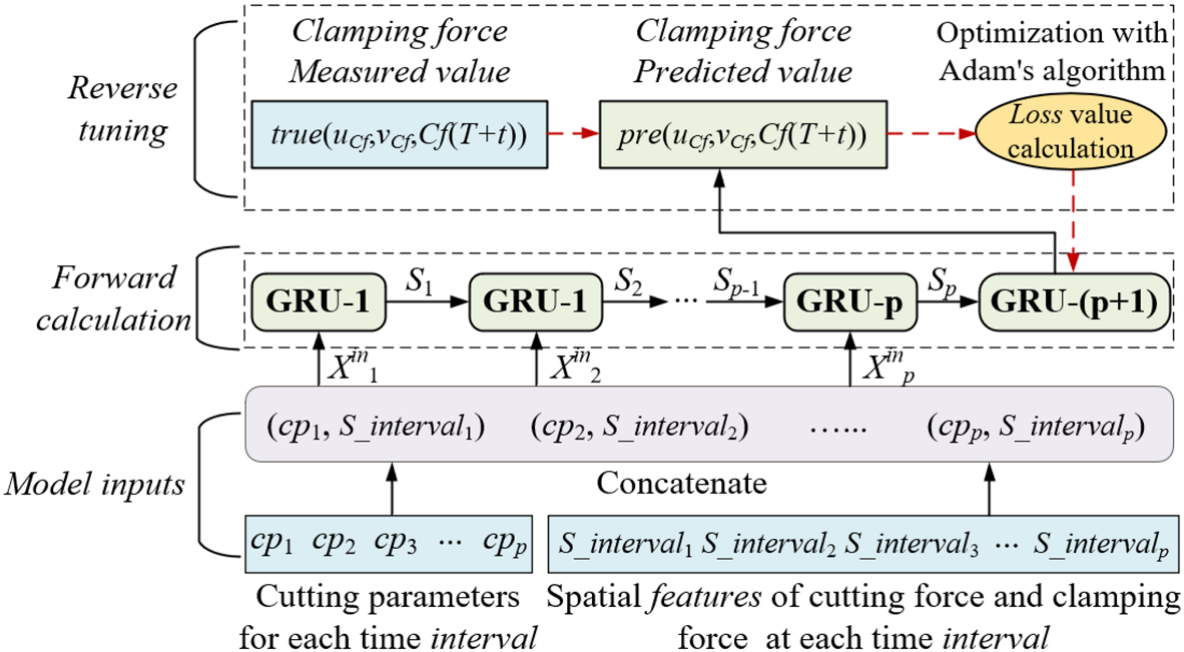
GRU network-based temporal correlation learning module.

To improve the learning efficiency of the prediction model, a concat algorithm is used to fuse the cutting parameters with the spatio-temporal correlation features in each sample, and the fused features are used as the input of the temporal learning model. Then, a GRU network is used to implement the learning of the temporal correlation between cutting force and clamping force and other data. The GRU network-based clamping force prediction model is shown in Fig. 6 below:

In the above figure, *cp*_*i*_ denotes the cutting parameters at different time intervals, and *S_interval*_*i*_ denotes the spatio-temporal correlation features at different time intervals after CNN extraction. Further, we have *X*_*i*_^*in*^ = (*cp*_*i*_, *S_interval*_*i*_). The bottom-up temporal correlation learning module consists of three main parts: module input, GRU network forward calculation, and prediction module inverse tuning.

The input of the temporal correlation learning module mainly uses the Concat algorithm to fuse the non-time-varying cutting parameters, and the spatial correlation features of cutting force and clamping force extracted by the CNN network, and then uses them as the input of the GRU network. Next is the forward calculation part of the GRU network, which mainly takes the fused features of each time interval of the current time period as the input, and outputs the clamping force prediction value at a future time after the forward calculation of the GRU network. Finally, the module is adjusted backwards, and we use the error back propagation principle to update and adjust the weights and biases of the neurons in the network. In this paper, the mean square error (MSE) is used as the loss function in the backward adjustment process, and the gradient descent process is optimized using the Adam algorithm.

### Joint training procedure

**Figure 7:**
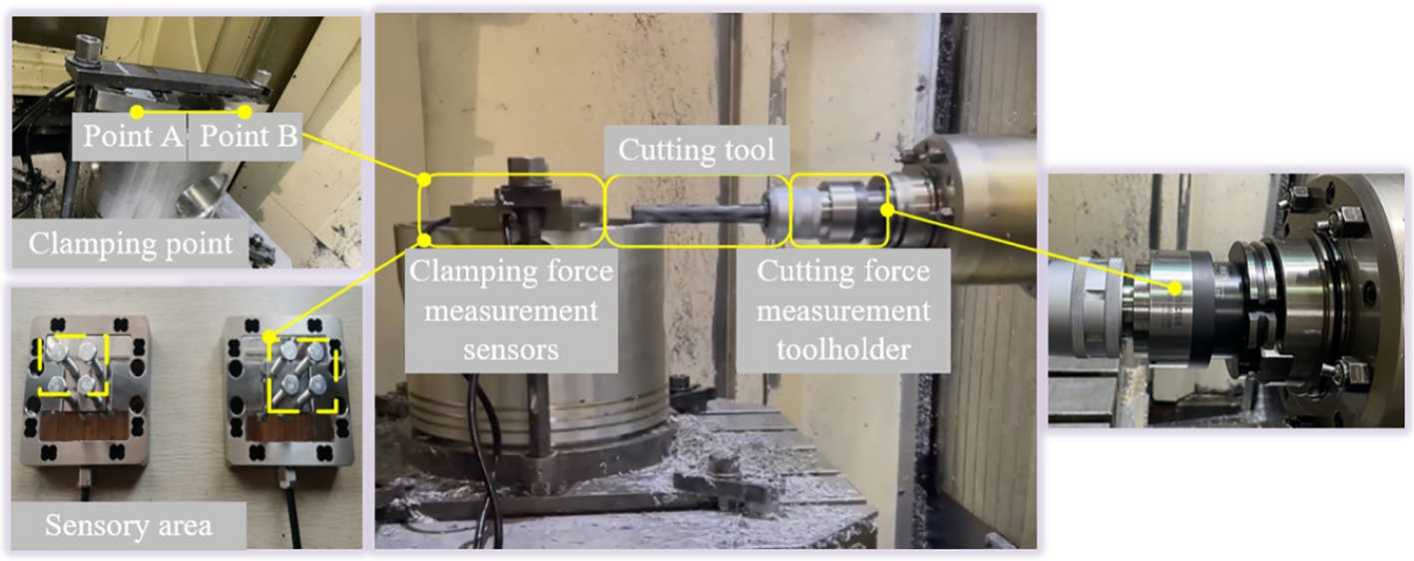
Machining experiment.

The convolutional neural network learns the spatial relationship between cutting force and clamping force at any time, and these spatial correlations at different times can be regarded as a time slice, and then the GRU network is used to model the temporal correlations between them to achieve the prediction of clamping force, so the two models are trained together here. The network structure used to learn the spatial relationship between cutting force and clamping force is mainly convolutional layer. For the convolutional layer, its input and output and the parameters to be trained can be expressed as:

11 $$ S\_interval = {\varvec{CNN}}(L){\kern 1pt} {\kern 1pt} $$

where *S_interval* denotes the spatio-temporal correlation features at different time intervals; ***CNN*** is the CNN network operator. Then, as shown in Fig. 7 above, the outputs of CNNs with different time intervals are concatenated with the corresponding cutting parameters to form the input of the GRU network. For example, $X_{1}^{in} = (cp_{1} ,S\_interval_{1} )$. Then rolling prediction is used to implement the learning of GRU network parameters.

12 $$ \begin{gathered} GRU(X_{1}^{in} ,X_{2}^{in} ,...,X_{t}^{in} ,...,X_{p}^{in} ){\kern 1pt} = {\kern 1pt} X_{p + 1}^{out} \hfill \\ GRU(X_{2}^{in} ,{\kern 1pt} X_{3}^{in} ...,X_{t}^{in} ,...,X_{p + 1}^{out} ){\kern 1pt} = {\kern 1pt} X_{p + 2}^{out} \hfill \\ GRU(X_{3}^{in} ,{\kern 1pt} X_{4}^{in} ...,X_{t}^{in} ,...,X_{p + 2}^{out} ){\kern 1pt} = {\kern 1pt} X_{p + 3}^{out} \hfill \\ ...... \hfill \\ \end{gathered} $$

That is, the clamping force of the *p* + 1 is predicted by inputting the relevant information of the previous* p* times, and then rolling repeatedly until the training set is completely covered, and then the training of the prediction model network is realized by continuously updating the input data. Specifically, The key to GRU is its recursive learning of temporal data, i.e., the output of the hidden layer contained in the network depends not only on the current input, but also on the output of the previous moment, and the process can be expressed as:

13 $$ S_{t} = f_{g} (W_{g} S_{t - 1} + UX_{t}^{in} ){\kern 1pt} {\kern 1pt} $$

where *S*_*t*_ denotes the output of the hidden layer at time *t*; *f*_*g*_ is an activation function of the hidden layer of the GRU network, *e.g.* the sigmoid *sigmoid*(*x*) = 1/(1 + *e*^*-x*^); *W*_*g*_, *U* are the learnable parameters in the hidden layer. Further, the above equation can be expanded as:

14 $$ \begin{gathered} X_{n}^{out} = g_{g} (VS_{t - 1} ) \hfill \\ {\kern 1pt} {\kern 1pt} {\kern 1pt} {\kern 1pt} {\kern 1pt} {\kern 1pt} {\kern 1pt} {\kern 1pt} {\kern 1pt} {\kern 1pt} {\kern 1pt} {\kern 1pt} {\kern 1pt} {\kern 1pt} {\kern 1pt} {\kern 1pt} {\kern 1pt} {\kern 1pt} {\kern 1pt} {\kern 1pt} = g_{g} (Vf_{g} (W_{g} S_{t - 1} + UX_{t}^{in} ){\kern 1pt} ) \hfill \\ {\kern 1pt} {\kern 1pt} {\kern 1pt} {\kern 1pt} {\kern 1pt} {\kern 1pt} {\kern 1pt} {\kern 1pt} {\kern 1pt} {\kern 1pt} {\kern 1pt} {\kern 1pt} {\kern 1pt} {\kern 1pt} {\kern 1pt} {\kern 1pt} {\kern 1pt} {\kern 1pt} {\kern 1pt} {\kern 1pt} = g_{g} (Vf_{g} (W_{g} (W_{g} S_{t - 2} + UX_{t - 1}^{in} ) + UX_{t}^{in} )) \hfill \\ {\kern 1pt} {\kern 1pt} {\kern 1pt} {\kern 1pt} {\kern 1pt} {\kern 1pt} {\kern 1pt} {\kern 1pt} {\kern 1pt} {\kern 1pt} {\kern 1pt} {\kern 1pt} {\kern 1pt} {\kern 1pt} {\kern 1pt} {\kern 1pt} {\kern 1pt} {\kern 1pt} {\kern 1pt} {\kern 1pt} ...... \hfill \\ \end{gathered} $$

where $X_{n}^{out}$ denotes the output of the GRU network at time *t*; *g*_*g*_ is the activation function of the output layer, e.g. hyperbolic tangent function tanh; *V* is the learnable parameter like *W*_*g*_ and *U*. Assuming that the actual value of clamping force at moment *t* is *X*_*n*_ and the output of prediction is $X_{n}^{out}$, the mean square error as the loss function of the whole prediction model can be expressed as:

15 $$ \begin{gathered} E_{t} = \frac{1}{2}(X_{n} - X_{n}^{out} )^{2} \hfill \\ E = \sum\limits_{t = 1}^{N} {E_{t} } \hfill \\ \end{gathered} $$

where *E*_*t*_ is the loss value at moment* t*, while *E* is the loss value for the entire training data *N*.

## Case study

### Data acquisition

In order to verify the validity of the proposed model, actual machining experiments are designed in this section and compared with other timing models and end-to-end models, respectively. Firstly, a cylindrical thin-walled piston skirt with forged aluminum alloy material is used as the validation object, and the key processes are shown in Table 1 below. Considering the clamping deformation during the actual milling of the piston skirt, this section collects the data related to the cutting force and clamping force during the machining of this piston skirt and verifies the proposed method by predicting the clamping force .

**Table 1:** Some key machining processes of piston skirt.

No	Name of the process
1	Forging stock
2	Rough turning of external circles, end faces, drilling
3	Scribing process
4	Rough Boring
5	Drilling, reaming
6	Semi-finish boring, milling of circular arcs

Compared with other machining processes, the arc milling process of the piston skirt has a higher material removal volume and does not require any changes to the existing fixture and its clamping method, which can avoid bringing uncertainties that affect the actual machining. Moreover, the clamping point of the milling process is closer to the position of the arc to be machined, and the force change at the clamping point is more obvious, so it is easy to collect the data of the force at this point. The specific experimental setup is shown in the Fig. 7 below.

For the cutting force and clamping force data, which are constantly changing with the cutting process, force sensors are needed to collect the corresponding data. Table 2 below shows the types of sensors and their sampling frequency.

**Table 2:** Data acquisition sensors.

Time-varying working condition signals	Sensor type	Sampling frequency (kHz)
Clamping force	Xinrui XR-D7	10
Cutting force	Spike toolholder	20

**Figure 8:**
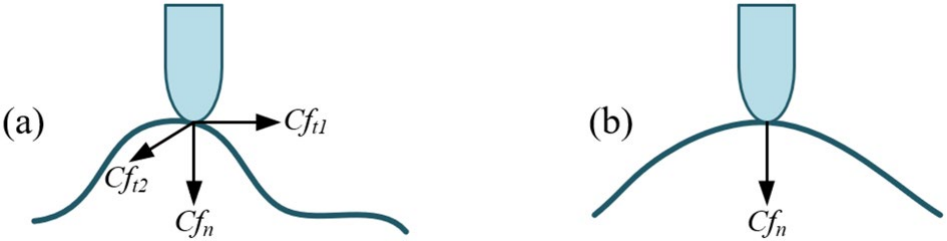
Common forms of part-fixture contact (**a**) normal clamping force—bidirectional friction contact and (**b**) normal clamping force contact.

Two common contact types between the part and the fixture are shown in the Fig. 8 below. In this paper, the clamping contact method shown in Fig. 8a is used, i.e., the normal-bidirectional friction contact method. More accurate results can be obtained by using a three-way force sensor to sense the change of clamping force. A three-way force sensor is used to sense the change of clamping force, which can obtain more accurate results. In the actual process monitoring, due to the geometry of the part and the limitations of the clamping form, it is often difficult to ensure that the sensor senses the direction of the part surface normal to the clamping point during the clamping process. It is difficult to obtain the exact position of the clamping force directly through the sensing method. However, in the actual experimental process, the sensor clamping surface used is small relative to the size of the entire part, and the clamping force position can be approximated as being in the middle of the clamping surface.

The cutting force data is collected by means of a SIPKE toolholder. The data collected by the toolholder can be transmitted to the computer via a wireless receiver. Since the toolholder collects the bending moment data in X/Y direction and the cutting force data in Z direction, we use the bending moment value data and the tool overhang length to calculate the cutting force data in X/Y direction. The milling cutter parameters are 4 edges, 8 mm diameter and 35 mm overhang. Cutting experiments were performed at four different sets of cutting parameters as shown in the Table 3 below:

**Table 3:** Milling process parameters.

Number	Spindle speed *n* (r/min)	Feed speed *v*_*f*_ (mm/min)	Cut depth *a*_*p*_ (mm)
C1	220	70	0.8
C2	250	80	1.0
C3	280	90	1.2
C4	220	80	1.2

### Data processing and model training

First, the sampling interval is set to ensure the synchronization of cutting force and clamping force in terms of data volume. Affected by the actual acquisition environment of the original signal and data acquisition equipment, there are usually missing data and data noise, which are likely to affect the subsequent prediction model construction results. In order to highlight the research content of this paper, the data in this paper have been pre-processed by pre-processing operations, including missing value processing, noise reduction and normalization, which will not be expanded in detail here. The input vector sequence of the clamping force prediction model is composed of input vectors corresponding to 200 time interval, and each input vector consists of three parts, namely cutting parameters, cutting force and spatial characteristics of the clamping force. The first set of cutting parameters in the Table 3 is used as an example to illustrate the process of obtaining the model inputs.

**Figure 9:**
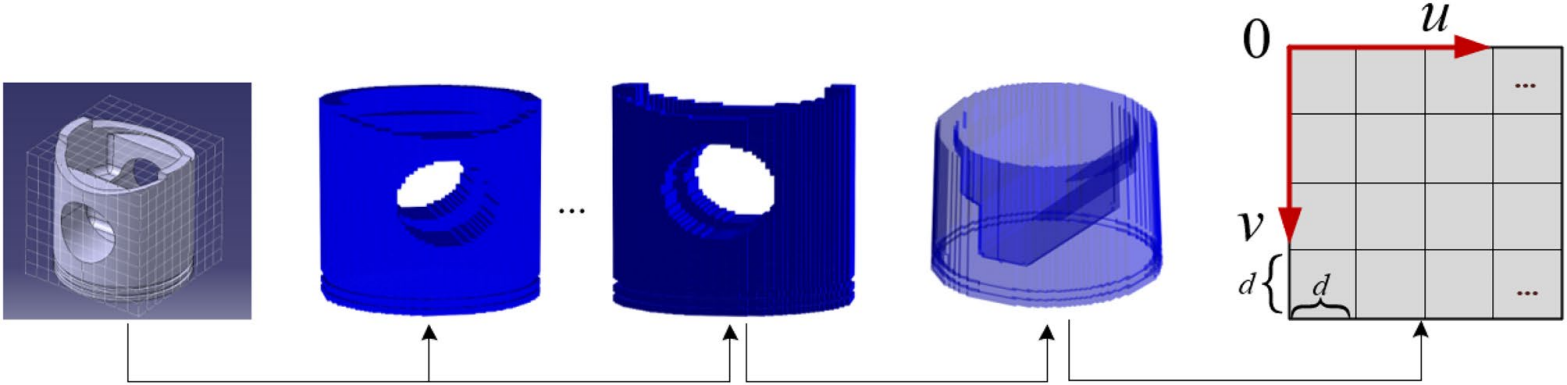
Part geometry parameterization.

First parameterize the part geometry model. For geometric information, if the voxel is inside the part, assign the voxel to + *d* which is the size of the voxel, if the voxel block is outside the part, assign the voxel to *-d*. Here, *d* indicates that the voxel block size is *d*mm × *d*mm × *d*mm. After that, a plane space where the cutting force and clamping force interact with each other is obtained from the angle of X–Y plane. This is mainly due to the fact that the actual cutting process is a smaller movement along the Z-direction, and the tool trajectory is easier to obtain in the X–Y plane, the clamping position and tool trajectory in the X–Y plane space changes relatively large. The piston skirt geometry parameterization process is shown in Fig. 9 below.

Using the parameterized part geometry as the basis for spatial information, and according to the spatio-temporal correlation operation in Sect. 4.2, the data set *L* is input to the CNN network force to obtain the spatial correlation features of both cutting force and clamping force. The input vector sequence of the clamping force prediction model consists of input vectors corresponding to 200 time interval, and each input vector consists of two parts, i.e., cutting parameters, and spatial correlation features of the two types of forces. The first set of cutting parameters in the table is used as an example to illustrate the process of obtaining the model inputs. Then the spatially correlated feature vectors corresponding to each time interval *interval*_1_ to *interval*_*200*_ are as follows:

$$ \begin{array}{*{20}l} {S\_interval_{{1}} = \left( { - {3}.{836}, \, - {4}.{662}, \, - {9}.{972}, \ldots , \, 0.{123}, \, - {7}.{986}, \, - {18}.{148}} \right)} \hfill \\ {S\_interval_{{2}} = \left( { - {21}.{212}, \, - {17}.{56}0,{ 2}.0{22}, \ldots , \, - {5}.{5}0{9},{ 1}.{8}00, \, - {3}.{569}} \right)} \hfill \\ \vdots \hfill \\ {S\_interval_{{{2}00}} = \left( { - {11}.{954}, \, - {15}.{299}, \, - {15}.{334}, \ldots , \, - {11}.{732}, \, - {2}0.{324}, \, - {3}.{766}} \right)} \hfill \\ \end{array} $$

Taking the first set of cutting parameters in Table 3 as an example, the cutting parameter vector *cp* can be denoted as follows:

$$ cp = \left( {n,v_{f} ,a_{p} } \right) = \left( {{ 22}0,{7}0,0.{8}} \right) $$

Then, the input of the prediction model for each time interval are obtained by fusing them using the Concat algorithm, as shown below.

$$ \begin{array}{*{20}l} {X^{in}_{{1}} = \left( {{2}0,{ 7}0, \, 0.{8}, \, - {3}.{836}, \, - {4}.{662}, \, - {9}.{972}, \ldots , \, - {27}.{855}, \, - {4}0{6}0.{582}} \right)} \hfill \\ {X^{in}_{{2}} = \left( {{22}0,{ 7}0, \, 0.{8}, \, - {21}.{212}, \, - {17}.{56}0,{ 2}.0{22}, \ldots , \, - {57}.{676}, \, - {4}0{29}.{679}} \right)} \hfill \\ {X^{in}_{{3}} = \left( {{22}0,{ 7}0, \, 0.{8}, \, - {8}.{155}, \, - {8}.{168}, \, - {16}.{938}, \ldots , \, - {75}.{9}0{4}, \, - {4117}.{677}} \right)} \hfill \\ \cdots \hfill \\ {X^{in}_{{{2}00}} = \left( {{22}0,{ 7}0, \, 0.{8}, \, - {11}.{954}, \, - {15}.{299}, \, - {15}.{334}, \ldots ,{ 17}.{575}, \, - {4}0{12}.{9}0{6}} \right)} \hfill \\ \end{array} $$

The length of the sample and the length of the time interval in this paper are 2 s and 0.01 s respectively, so the constructed GRU network contains a total of 201 GRU structural units after being expanded along the time axis, where the first 200 unit structures are input into the fusion vector corresponding to 1 × 585 in the time interval, and finally the clamping force state prediction is output in the 201st structural unit.

**Figure 10:**
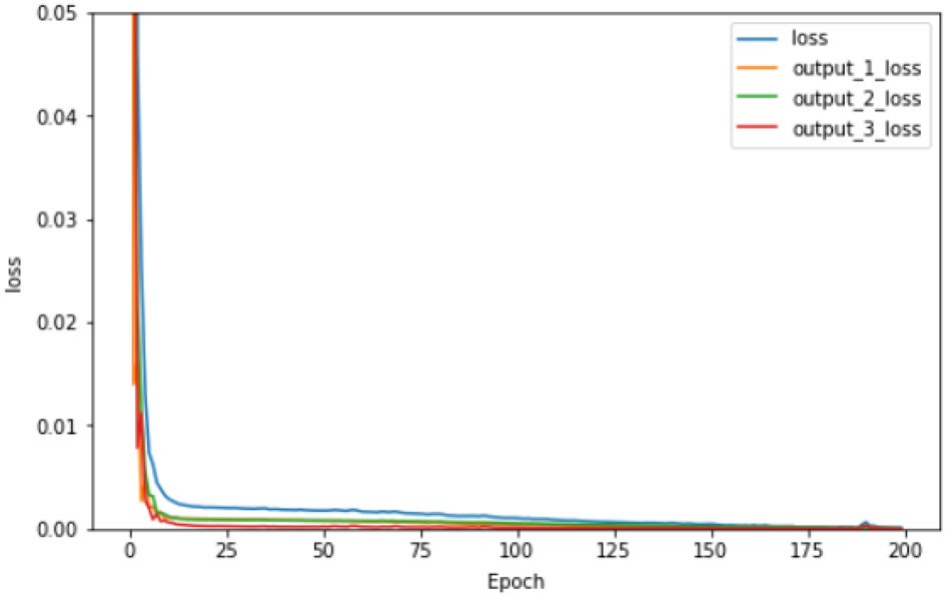
The variation curves of loss values.

The model training process is set with a learning rate of 0.0015, a batch size of 128, and a training iteration number of 200, and the gradient descent process is optimized using the Adam algorithm. The variation curves of the loss function (MSE values) of the training process of the prediction model under the cutting parameters of Table 3 are shown in Fig. 10.

### Result and discussion

**Figure 11:**
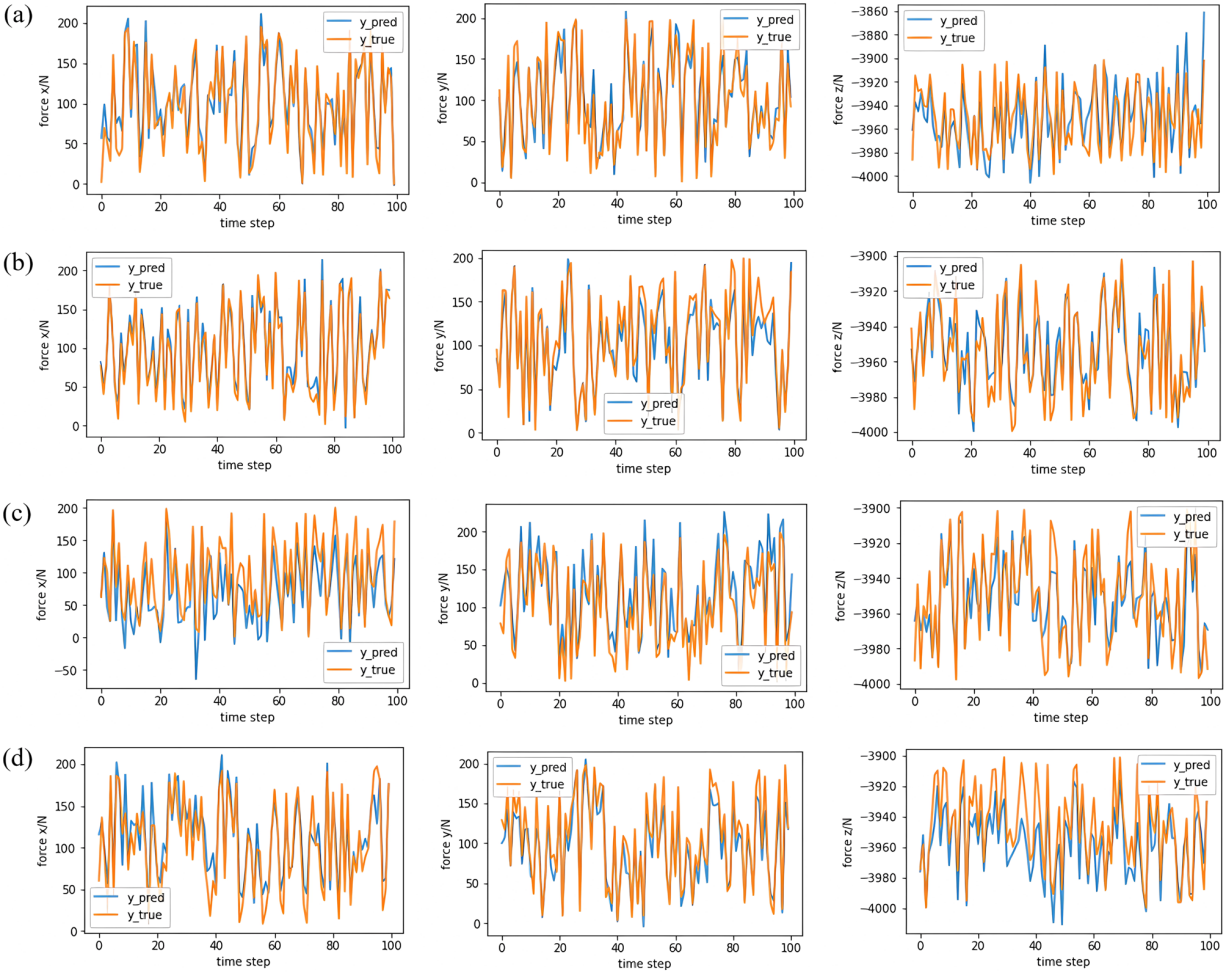
The prediction results of the proposed model under cutting parameters C1 (**a**)–C4 (**d**).

The fitting of the predicted results of the clamping force for four cutting parameters to the real measurement results is shown in Fig. 11.

Based on the above prediction model, we used the cross-validation method to verify the generalization of the clamping force prediction model. First, cross validation schemes are designed based on the cutting parameters in Table 3, as shown in Table 4 below.

**Table 4:** Cross-validation schemes.

Scheme No	Training set	Test set
1	C2, C3, C4	C1
2	C1, C3, C4	C2
3	C1, C2, C4	C3
4	C1, C2, C3	C4

To quantify the performance of the proposed model, the mean absolute error (MAE) and root mean square error (RMSE) are used here. The indicators for each validation scheme are shown in the Table 5 below.

**Table 5:** Measurement indicator (MAE and RMSE).

Scheme No	MAE	RMSE
1	0.16	0.19
2	0.23	0.29
3	0.17	0.20
4	0.16	0.20

**Figure 12:**
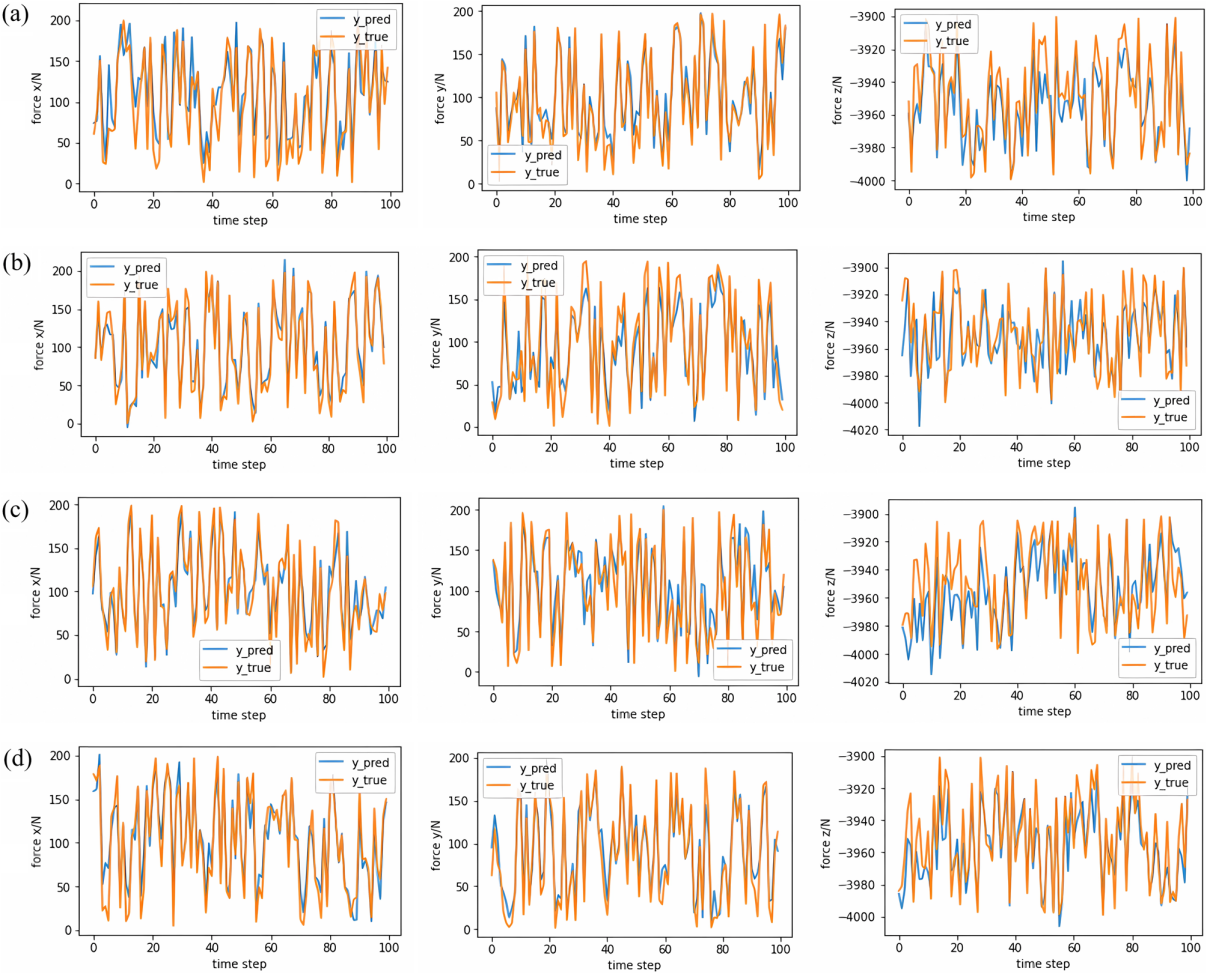
The prediction results of the proposed model under cross-validation schemes 1 (**a**)–4 (**d**).

It can be seen that the regression error of the model proposed in this paper on the validation schemes fluctuates, but in general exhibits a low regression error. Further, the model prediction results are compared with the actual measurement results as shown in the following Fig. 12.

It can be seen from the above figure that the predicted results are relatively accurate. There may be two reasons for the fluctuations, one is that the contact point between the sensor and the part is not exactly perpendicular to the surface of the part, and the force collected is not the ideal clamping position; the second is that the initial clamping force in the Z-direction is larger, and the sensitivity to changes in the clamping force is not as acute as in the other two directions, thus causing the prediction results to fluctuate more as well.

**Figure 13:**
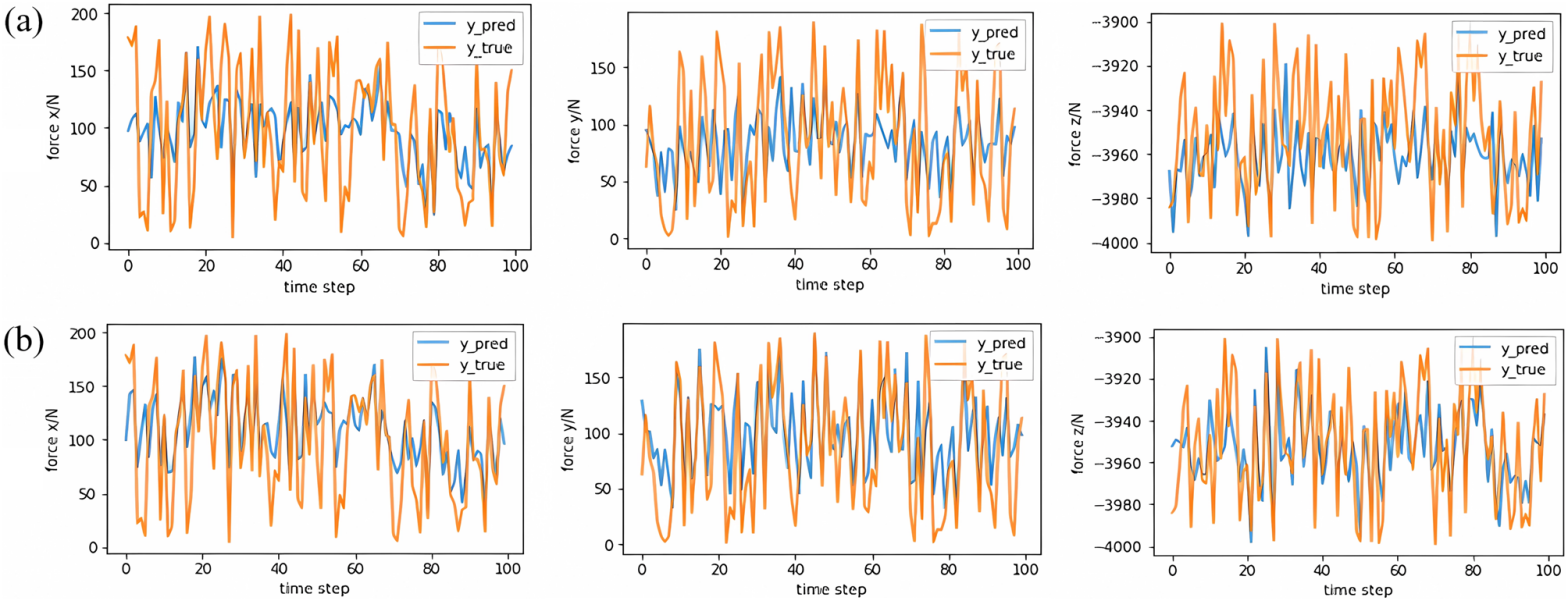
(**a**) CNN-RNN model prediction result. (**b**) CNN-BP model prediction result.

Further comparison. To demonstrate the advantages of the proposed models, CNN-RNN models and CNN-BP models were trained using the second set of cutting parameter data in Table 3. The results of each prediction model are shown in Fig. 13 below.

Further, we chose root mean square error (RMSE) and mean absolute error (MAE), as assessment metrics to quantitatively compare the predictive ability of the four models. The calculated results of the mean values of the evaluation indexes for each model are shown in the following Table 6.

**Table 6:** Comparison of measurement indicators.

	CNN-GRU	CNN-RNN	CNN-BP
RSME	0.29	0.61	0.56
MAE	0.23	0.64	0.52

Then, the RMSE and MAE are continued to be used as measurement indicators to quantitatively compare the predictive ability of different models. The results of the measurement indicators for each model are shown in Table 6 below.

It can be seen that the proposed model has the smallest RMSE and MAE values of 0.29 and 0.23, respectively, which means that the proposed model has better prediction ability compared with the CNN-RNN model and the CNN-BP model.

## Conclusion

Accurate prediction of the the clamping force of thin-walled parts is an effective way to optimize fixture clamping and further suppress machining distortion. Considering that the clamping force is influenced by the clamping position, and the combined effect of cutting force and other factors. In this paper, the interaction between cutting force and clamping force is considered as a spatio-temporal correlation between the two acting together on the part geometry space and in dynamic equilibrium, and a clamping force prediction method based on a deep spatio-temporal network is proposed. The parameterized part geometry model is used as the basis of spatial correlation between cutting force and clamping force, and the spatio-temporal correlation between cutting force and clamping force is performed; then, the full convolutional neural network is designed to model the spatial correlation relationship between cutting force and clamping force; after that, the spatial correlation features at different times are regarded as time slices, and the GRU network is used to model the temporal correlation relationship between the two to achieve the prediction of clamping force. Finally, experimental validation is carried out using thin-walled cylindrical parts, and the results prove the effectiveness of the prediction method proposed in this paper.

Compared to cutting forces, clamping forces are relatively difficult to measure in real machining scenarios, mainly due to the complex geometric features of the part, as well as the limitations of the clamping equipment and clamping positions. However, it is not easy to obtain the spatial correlation between cutting force and clamping force for complex parts, which may also affect the accuracy of the method in this paper to predict the clamping force. Also, the location of the clamping force measurement is one of the factors that affect the accuracy of the model. Further, this paper only predicts the force state of key clamping points during the machining process, while the deformation of the part is the result of the overall force on the part. Therefore, how to extend the proposed method of force prediction at clamping points to force prediction at any point on the part and the prediction of the overall force distribution of the part is the future research content.

## Data Availability

The datasets generated and/or analysed during the current study are not publicly available due the data also forms part of an ongoing study but are available from the corresponding author on reasonable request.
